# Comparative risk of infections among real-world users of biologics for juvenile idiopathic arthritis: data from the German BIKER registry

**DOI:** 10.1007/s00296-020-04774-3

**Published:** 2021-02-16

**Authors:** Franz Thiele, Ariane Klein, Daniel Windschall, Anton Hospach, Ivan Foeldvari, Kirsten Minden, Frank Weller-Heinemann, Gerd Horneff

**Affiliations:** 1 Centre for Paediatric Rheumatology, Department of General Paediatrics, Asklepios Clinic Sankt Augustin, 53757 Sankt Augustin, Germany; 2 Medical Faculty, University of Cologne, Cologne, Germany; 3 Clinic for Paediatric and Adolescent Rheumatology, St. Josef-Stift Sendenhorst, Northwest German Center for Rheumatology, Sendenhorst, Germany; 4 Centre for Paediatric Rheumatology, Klinikum Stuttgart, Germany; 5 Hamburger Zentrum Für Kinder- Und Jugendrheumatologie, Hamburg, Germany; 6 Charité University Medicine Berlin, Berlin, Germany; 7 Division of Paediatric Rheumatology, Prof. Hess Children’s Hospital, Bremen, Germany

**Keywords:** Juvenile idiopathic arthritis, Safety, Biologics, Infections

## Abstract

To examine whether treatment with interleukin (IL)-1-, IL-6-, tumour necrosis factor α (TNFα)-inhibitors or Abatacept is associated with an increased risk of common infections, infections requiring hospitalization (SAE) or opportunistic infections among real-world juvenile idiopathic arthritis (JIA) patients. Furthermore, the influence of other patient-related covariates on the occurrence of infections was investigated. Patients diagnosed with JIA and treated with biologics were selected from the German BIKER registry. Incidence rates (IR) of infections per 100 person years were calculated and compared between the different cohorts. Using multivariate logistic regression, odds ratios with 95% confidence intervals (CI) were determined for the influence of patient-related covariates (age, diagnosis, laboratory data, concomitant medication, JIA activity, comorbidities, and premedication) on the occurrence of infections. 3258 patients entered the analysis. A total of 3654 treatment episodes were distributed among TNFα- (Etanercept, Adalimumab, Golimumab, Infliximab, *n* = 3044), IL-1- (Anakinra, Canakinumab, *n* = 105), IL-6- (Tocilizumab, *n* = 400) and T-cell activation inhibitors (Abatacept, *n* = 105). 813 (22.2%) patients had at least one infection, 103 (2.8%) patients suffered from an SAE infection. Both common and SAE infections were significantly more frequent in IL-1 (IR 17.3, 95% CI 12.5/24 and IR 4.3, 95% CI 2.3/8.3) and IL-6 cohort (IR 16.7, 95% CI 13.9/20 and IR 2.8, 95% CI 1.8/4.4) compared to TNFα-inhibitor cohort (IR 8.7, 95% CI 8.1/9.4 and IR 1, 95% CI 0.8/1.3). When comparing the influencing factors for various infectious diseases, the use of corticosteroids, younger age, cardiac comorbidities and higher JIA-activity are the most striking risk factors. Relative to TNFα inhibitors and Abatacept, IL-1 and IL-6 inhibitors were associated with an increased risk of common and SAE infections. The influencing covariates identified may be helpful for the choice of a suitable biologic to treat JIA.

**Supplementary Information:**

The online version contains supplementary material available at 10.1007/s00296-020-04774-3.

## Introduction

Biologics play an important role in the treatment of juvenile idiopathic arthritis (JIA). JIA is the most common rheumatic disease in children and can lead to severe joint destruction [1]. Depending on the JIA category, various biologics are approved. Currently, three tumour necrosis factor α-inhibitors (TNFi) Etanercept (ETA), Adalimumab (ADA) and Golimumab (GOL) as well as Abatacept (ABA), an inhibitor of T-cell activation, are approved for different subtypes of non-systemic JIA [2]. The first developed TNFi Infliximab (INF) is also used to treat non-systemic JIA, although it is not approved in this indication. Tocilizumab (TOC), an inhibitor of Interleukin-6 (IL-6i), is approved for both polyarticular (pJIA) and systemic JIA (sJIA). Additionally, Anakinra (ANA) and Canakinumab (CAN) are approved for treatment of sJIA [3]. Both biologics are inhibitors of Interleukin-1 (IL-1i). Treatments with biologics may be continued for years, so data on safety should be considered. Especially for detection of severe and rare adverse events (AE), a large quantity of patient years is necessary. The German BIKER registry, on which this analysis is based, has such large patient numbers with prolonged observation time.

Infections during treatment of JIA with biologics are one of the most frequent occurring AE [4]. Among these infections that may be associated with biologics are various infectious diseases, including both common viral respiratory infections and serious bacterial infections [1]. All biologics used to treat JIA are suspected of contributing to an increased risk of infection through their immunosuppressive effect [5]. Furthermore, more than half of all JIA patients treated with biologics receive an additional immunosuppressive medication, mostly Methotrexate [6].

Placebo-controlled randomized trials (RCTs) regarding ETA, ADA and GOL showed no increased number of infections during treatment of non-systemic JIA [7–9]. For treatment of sJIA, use of TOC was associated with an increased risk of infections in a RCT [10]. Despite these reports, gaps in our comprehension of this entity remain.

In this prospective observational study, we aimed to add important information regarding the understanding whether these findings from RCTs persist in real-world practice, where patients are more heterogeneous and drug utilisation is far less controlled. One task of this study is to determine the risk of infections, especially serious infections that may require hospitalization. In addition to a comparison of infection rates among biologics with different molecular targets, this study also examines other influencing factors that could affect the occurrence of various infectious diseases in patients with JIA treated with biologics.

## Methods

### Study population and ethics statement

The German BIKER registry is a prospective, observational registry, which has already been described in previous reports [11, 12]. BIKER is an acronym in German and stands for Biologics in pediatric rheumatology. Since 2001, JIA therapies with biologics have been documented, which corresponds to about 5000 observed patients. BIKER was approved by the local ethics committee of the Aerztekammer Nordrhein, Duesseldorf, Germany, reference number 2/2015/7441. Written informed consent was obtained and pseudonymized data were collected for each patient. Patient assessment was performed at baseline, after 3 and 6 months and every 6 months thereafter.

### Inclusion and exclusion criteria

Patients diagnosed with JIA and treated with ABA, ADA, ANA, CAN, ETA, GOL, INF or TOC were selected for this analysis. All TNFi (ADA, ETA, GOL, INF) were grouped for various comparisons, the same applies for IL-1i (ANA, CAN). If a patient received several biologics from the same group (only possible in the TNFi or IL-1i group), this was evaluated as one treatment episode. Patients could also receive biologics from different groups, which were then evaluated as separate treatment episodes. These treatment episodes have been included in this analysis. Methodological effects seem negligible due to the low number of patients (*n* = 339) who contributed to two analysis cohorts.

**Fig. 1:**
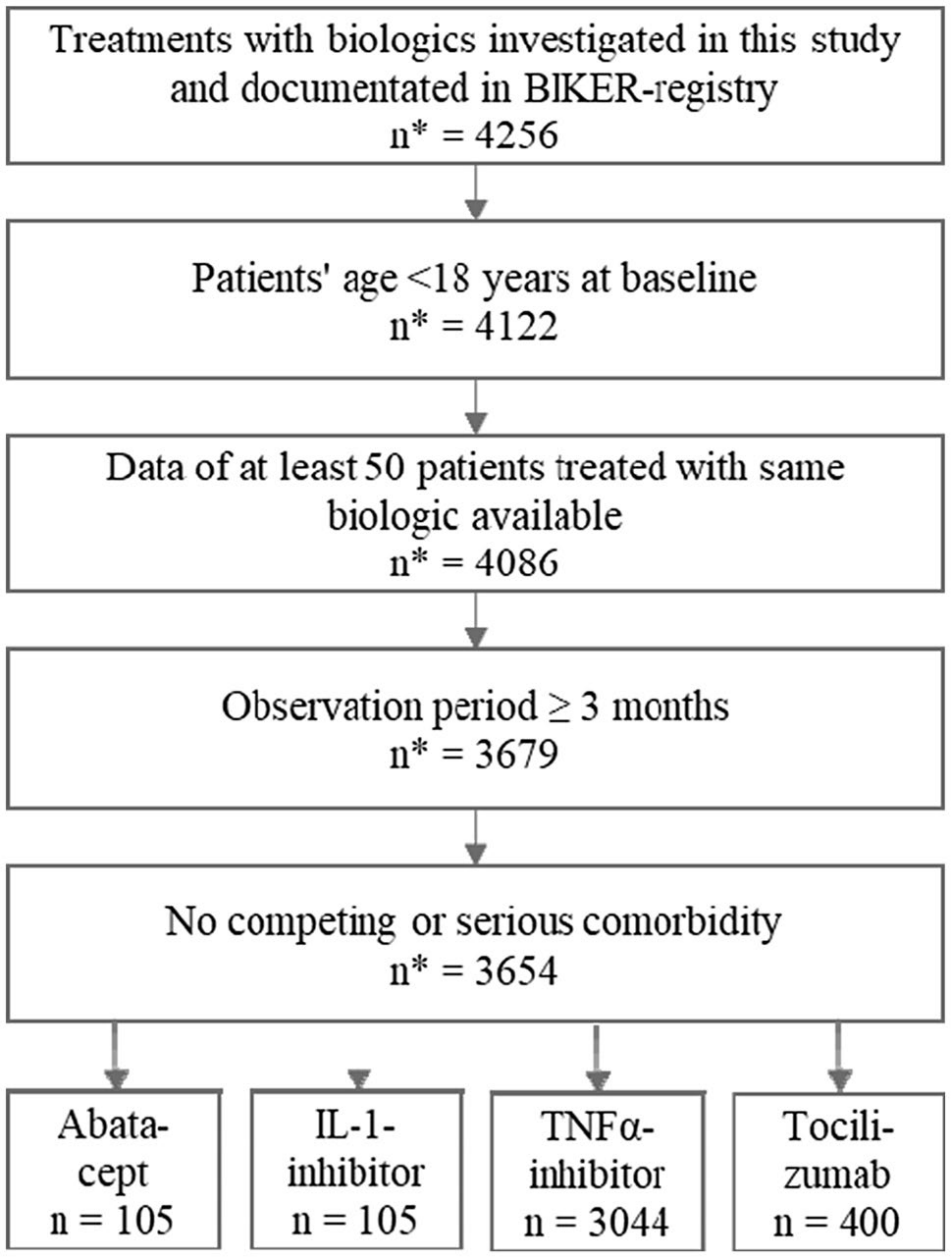
Patient selection process from the BIKER registry according to the inclusion and exclusion criteria of our study. *IL* interleukin, *TNF* tumour necrosis factor, *number of treatment episodes

Diagnosis of severe rheumatoid diseases except JIA (e.g. sarcoidosis, SLE, Behçet disease), inflammatory bowel diseases or malignancies were classified as competing comorbidities due to their potential impact on the incidence especially of serious infections [13]. Data documented from the beginning of the registry on January 1, 2001 to March 1, 2020 were included. The patient selection progress is shown in Fig. 1.

### Safety analyses and definitions

Analysis regarding safety was based on AE reports. According to ICH E6 section 1.2 [14], an AE is any untoward medical occurrence in a subject temporarily associated with a pharmaceutical product, even without causality or relationship. This analysis focused on infections, so we basically considered various infectious AE of included patients. Serious adverse events (SAE) included death, a life-threatening event, an event leading to or prolonging hospitalization, persistent or significant disability/incapacity or an important medical event requiring medical or surgical intervention to prevent a serious outcome or congenital anomaly or birth defect. All cases of Herpes zoster, pneumonia, and varicella were classified as adverse events of special interest (AESI). Opportunistic infections were classified as described by Winthrop et al. [15]. AE were requested and documented at every visit. In addition, patients and treating physicians had the possibility to report AE directly at any time. The MedDRA system [16] was used to categorize the AE reports. Any infections were assigned to a therapy if it occurred during treatment or up to 90 days after discontinuation. In case another biologic with a different molecular target was started during this period of 90 days, the infection was also counted for the new therapy. For each entity of infectious diseases (non-serious AE, SAE, each AESI), only the first occurrence under therapy with the respective biologic group was considered.

We have investigated the influence of multiple covariates known at baseline of a treatment episode on the occurrence of infections. Patient covariates included demographics (age, sex), body weight and length, body mass index (BMI), diagnosis, exposure time to treatment with biologic, laboratory data (positivity of antinuclear antibodies, human leukocyte antigen B27, anti-citrullinated protein antibodies), concomitant medication at baseline, various parameters of JIA-activity at baseline, premedication and comorbidities. Several parameters on the disease activity of JIA are also part of the Juvenile Arthritis Multidimensional Assessment Report (JAMAR), which was developed by the Paediatric Rheumatology International Trials Organisation [17, 18]. The disease activity of JIA was furthermore quantified using the Juvenile Arthritis Disease Activity Score (JADAS-10) [19].

### Statistical analysis

Statistical analyses show frequencies, incidence rates (IR) per 100 patient years and odds ratio (OR), both with 95% confidence interval (CI).

The Chi-square test and Wald test were used to compare the infection frequencies in the cohorts. The influence of the covariates mentioned above was on the one hand determined by univariate comparison between patients affected by an infection and non-affected patients. The univariate comparisons for categorical variables were performed using the Chi-square test, for continuous non-normally distributed variables the Mann–Whitney *U* test was used. On the other hand, we have performed multivariate logistic regressions to adjust the interdependencies of the covariates and to create a predictive model for infections based on patient characteristics known at baseline of a treatment episode. For continuous variables, we have determined the OR for a defined number of units in both the univariate and multivariate approaches. For each continuous variable, the number of units to which the OR refers is indicated separately in the result section.

Significance level was set at 5%, analyses were performed by SPSS version 25 (IBM).

## Results

### Patient characteristics

We identified a total of 3258 patients with 3654 treatment episodes. 95% of patients with ethnicity information had a Caucasian ethnicity, the remaining 5% divided between African and Asian ethnicities. Patient characteristics at baseline are shown in Table 1. With 2523 treatments, ETA was the most frequently used drug in the TNFi-cohort, followed by ADA (976 treatments), GOL (132 treatments) and INF (64 treatments). The IL-1i-cohort consists of ANA and CAN (63 and 61 treatments). When looking at the patient characteristics, several differences between the cohorts treated with different biologics are noticeable (Table 1). With regard to the comorbidities, it should be added that the most common cardiac comorbidity was mitral valve disease. Bronchial asthma was the most frequent disease among the respiratory comorbidities, atopic dermatitis among the dermatological comorbidities and uveitis among the eye disorders.

**Table 1:** Characteristics of patients at baseline, overall and by drug class

Drug class	TNF-α-inhibitors	Tocilizumab	Interleukin-1-inhibitors	Abatacept	All
Treatment episodes	*n* = 3044	*n* = 400	*n* = 105	*n* = 105	*n* = 3654
Age at baseline	12.3 [8.6/15.2]	12.3 [8.8/15]	9.1 [4.8/13.3]**	14.2 [11.2/16.3]*	12.3 [8.6/15.2]
Female sex	2059 (67.6%)	302 (75.5%)*	41 (39%)**	88 (83.8%)*	2490 (68.1%)
Diagnosis					
Systemic arthritis	127 (4.2%)**	108 (27%)*	99 (94.3%)*	5 (4.8%)	339 (9.3%)
Polyarticular arthritis, RF-	1019 (33.5%)	158 (39.5%)*	4 (3.8%)**	46 (43.8%)*	1227 (33.6%)
Polyarticular arthritis, RF +	220 (7.2%)	30 (7.5%)	0**	10 (9.5%)	260 (7.1%)
Oligoarthritis	833 (27.4%)*	81 (20.3%)**	1 (1%)**	30 (28.6%)	945 (25.9%)
Psoriatic arthritis	200 (6.6%)*	8 (2%)**	0**	8 (7.6%)	216 (5.9%)
Enthesitis-related arthritis	545 (17.9%)*	5 (1.3%)**	0**	5 (4.8%)**	555 (15.2%)
Undifferentiated arthritis	100 (3.3%)	10 (2.5%)	1 (1%)	1 (1%)	112 (3.1%)
Antinuclear antibodies (ANA)	1536 (50.5%)*	192 (48%)	11 (10.5%)**	52 (49.5%)	1791 (49%)
JADAS-10 at baseline	13.9 [8.9/19.3]	14.6 [9/20.2]	12.7 [6.4/19.4]	12.6 [8.5/16.9]	13.9 [8.9/19.3]
Number of previous biologics					
First-line therapy	2923 (96%)*	101 (25.3%)**	41 (39%)**	8 (7.6%)**	3073 (84.1%)
Second-line therapy	94 (3.1%)**	156 (39%)*	54 (51.4%)*	28 (26.7%)*	332 (9.1%)
Third or higher line therapy	27 (0.9%)**	143 (35.8%)*	10 (9.5%)	69 (65.7%)*	249 (6.8%)
MTX pretreatment	2638 (86.7%)*	354 (88.5%)	57 (54.3%)**	98 (93.3%)*	3147 (86.1%)
Other DMARDs pretreatment	788 (25.9%)	73 (18.3%)**	24 (22.9%)	39 (37.1%)*	924 (25.3%)
NSAIDs pretreatment	2703 (88.8%)	347 (86.8%)	84 (80%)**	101 (96.2%)*	3235 (88.5%)
Corticosteroids pretreatment	1560 (51.2%)**	295 (73.8%)*	84 (80%)*	83 (79%)*	2022 (55.3%)
Number of comorbidities					
Eye disorders	370 (12.2%)	53 (13.3%)	1 (1%)**	14 (13.3%)	438 (12%)
Respiratory disorders	71 (2.3%)	7 (1.8%)	2 (1.9%)	4 (3.8%)	84 (2.3%)
Cardiac disorders	36 (1.2%)**	10 (2.5%)	7 (6.7%)*	0	53 (1.5%)
Dermatologic disorders	114 (3.7%)	14 (3.5%)	2 (1.9%)	3 (2.9%)	133 (3.6%)

Continuous variables are presented as median [25% and 75% quartile], categorical variables are presented as counts (percentages)

Other DMARDs pretreatment means treatment with at least one of azathioprine, chloroquine, ciclosporin A, leflunomide, sulfasalazine

*RF* rheumatoid factor, *DMARD* disease modifying antirheumatic drug, *NSAID* non-steroidal anti-inflammatory drug, *MTX* methotrexate, *JADAS* Juvenile arthritis disease activity score, *TNF* tumour necrosis factor

*Value in cohort significantly higher

**Value in cohort significantly lower compared to all other cohorts

### Rate of infections

Altogether, 1614 infections were reported. In 813 treatment episodes (22.2%) at least one infection occurred. The median time between the start of a biologic and the occurrence of an infections was 8 months (25% and 75% quartile: 3 and 20 months). 103 (2.8%) patients were affected by a SAE infection. In Table 2, the numbers and incidence rates of all infections, infections fulfilling SAE criteria and infections of special interest (AESI) are given. Several significant differences were noted as outlined in Table 2. In this table, we also compared the rates of all and SAE infections in the different cohorts when only considering patients with sJIA.

**Table 2:** Incidence of infections among all biologic users and biologic users with systemic arthritis, overall and by drug class

	TNFi	Tocilizumab	IL-1i	Abatacept	All
Drug class					
Number of treatment episodes	*n* = 3044	*n* = 400	*n* = 105	*n* = 105	*n* = 3654
Total person years of follow-up	7377.8	677.87	207.52	145.07	8408.25
Incident infections, *n* (%)	643 (21.1%)	113 (28.2%)**	36 (34.3%)**	21 (20%)	813 (22.2%)
Incidence rate°	8.7 [8.1/9.4]*	16.7 [13.9/20]***	17.3 [12.5/24]***	14.5 [9.4/22]	9.7 [9/10.4]
Incident SAE infections, *n* (%)	75 (2.5%)	19 (4.8%)*	9 (8.6%)**	0	103 (2.8%)
Incidence rate°	1 [0.8/1.3]	2.8 [1.8/4.4]***	4.3 [2.3/8.3]***	0	1.2 [1/1.5]
Herpes zoster, *n* (%)	31 (1%)	3 (0.8%)	0	0	34 (0.9%)
Incidence rate°	0.4 [0.3/0.6]	0.4 [0.1/1.4]	0	0	0.4 [0.3/0.6]
Pneumonia, *n* (%)	22 (0.7%)	6 (1.5%)	2 (1.9%)	0	30 (0.8%)
Incidence rate°	0.3 [0.2/0.5]	0.9 [0.4/2]*	1 [0.2/3.9]	0	0.4 [0.2/0.5]
Varicella, *n* (%)	21 (0.7%)	1 (0.3%)	1 (1%)	0	23 (0.6%)
Incidence rate°	0.3 [0.2/0.4]	0.2 [0.02/1.1]	0.5 [0.1/3.4]	0	0.3 [0.2/0.4]
Only considering patients with systemic juvenile idiopathic arthritis					
Number of treatment episodes	*n* = 127	*n* = 108	*n* = 99	*n* = 5	*n* = 339
Total person years of follow-up	336.34	233.09	195.43	5.54	770.4
Incident infections, *n* (%)	14 (11%)	41 (38%)***	34 (34.3)***	1 (20%)	90 (26.5%)***
Incidence rate°	4.2 [2.5/7]	17.6 [13/23.9]***	17.4 [12.4/24.3]***	18.1 [2.5/128]	11.7 [9.5/14.4]***
Incident SAE infections, *n* (%)	3 (2.4%)	14 (13%)**	9 (9.1%)*	0	26 (7.7%)*
Incidence rate°	0.9 [0.3/2.8]	6 [3.6/10.1]**	4.6 [2.4/8.9]*	0	3.4 [2.3/5]*

*TNFi* tumour necrosis factor-α-inhibitors, *IL-1i* Interleukin-1-inhibitors, *SAE infections* infections requiring hospitalization. *°* Incidence rate [95% confidence interval], per 100 person years

*p < 0.05 vs all, **p < 0.01 vs all, ***p < 0.001 vs all. For comparisons of only patients with systemic arthritis, *p* is vs TNFi, not vs all

Significantly more infections were reported in patients treated with IL-1i (IR 17.3, 95% CI 12.5/24) or IL-6i (IR 16.7, 95% CI 13.9/20) than in patients treated with TNFi (IR 8.7, 95% CI 8.1/9.4). Infections classified as SAE also occurred more frequently in these two cohorts. The most occurring entities among the common and SAE infections are shown in Table 3. A comparison of the cohorts by different age groups showed that these significant clusters were particularly pronounced among children of pre-school age. The significant differences remained when only patients with sJIA were considered. It should be noted that patients who received a TNFi to treat sJIA were more likely to receive corticosteroids at baseline and had no lower disease activity than patients with sJIA in the IL-1i- or TOC-cohort.

**Table 3:** Most frequent disease entities in our analysis, regarding all infections and infections requiring hospitalization (SAE)

LLT	Frequency	LLT	Frequency
Incident infections (all)	813	Incident SAE infections	103
Upper respiratory tract infection	412	Pneumonia	11
Tonsillitis	67	Upper respiratory tract infection	10
Gastroenteritis	63	Abscess	9
Urinary tract infection	32	Urinary tract infection	9
Otitis media	28	Tonsillitis	9
Streptococcal infection	20	Gastroenteritis	7

*LLT* Lowest level terms

In contrast, Herpes zoster and varicella occurred numerically predominant in patients treated with TNFi. Besides Herpes zoster, opportunistic infections were rare. No cases of active tuberculosis occurred, two patients suffered from oral candidiasis.

### Covariates

To examine the impact of covariates, we performed univariate comparisons between patients affected by any, SAE or AESI infections and non-affected patients. All univariate tested covariates are listed in supplementary Table 1. The main results of these univariate comparisons are presented in the following three sections:

Patients treated with IL-1i (OR 1.9, 95% CI 1.2–2.8) or IL-6i (OR 1.4, 95% CI 1.1–1.8) had a higher rate of any infections. Same goes for diagnosis of sJIA (OR 1.3, 95% CI 1–1.7), cardiac comorbidities (OR 2.5, 95% CI 1.5–4.4) and premedication with corticosteroids (OR 1.3, 95% CI 1.1–1.5). The older the patient at baseline, the lower was the risk of infection (OR per 5 years 0.6, 95% CI 0.5–0.64). High disease activity of JIA at baseline was only slightly associated with the occurrence of an infection.

Treatment with IL-1i (OR 3.4, 95% CI 1.7–7) or IL-6i (OR 1.9, 95% CI 1.1–3.1) was associated with a more frequent occurrence of SAE infections. Patients with diagnosed sJIA (OR 3.5, 95% CI 2.2–5.5), having a comedication with corticosteroids at baseline (OR 2.3, 95% CI 1.5–3.4) or having cardiac comorbidities (OR 3.7, 95% CI 1.5–9.6) also had an increased risk regarding serious infections. Older patients at baseline were less frequently affected by SAE infections (OR per 5 years older 0.5, 95% CI 0.4–0.7). Numerous parameters concerning the disease activity of JIA indicate an association of increased disease activity and the occurrence of SAE infections, e.g. JADAS-10 (OR per 10 index units 1.6, 95% CI 1.2–2.2).

Intake of corticosteroids at baseline (OR 2, 95% CI 1–4), cardiac comorbidities (OR 4.4, 95% CI 1–18.7), young age at disease onset (OR per 5 years older 0.7, 95% CI 0.5–1) and increased disease activity of JIA as measured by JADAS-10 at baseline (OR 1.9, 95% CI 1.2–3.1) are risk factors for Herpes zoster.

In contrast, the disease activity of JIA is insignificant for the risk of pneumonia. Diagnosis of sJIA (OR 3.6, 95% CI 1.6–8.2) and younger age at baseline (OR per 5 years older 0.5, 95% CI 0.4–0.8) increases the chance of pneumonia. Regarding a primary varicella infection, use of corticosteroids at baseline (OR 2.4, 95% CI 1–5.4), younger age at baseline (OR per 5 years older 0.3, 95% CI 0.2–0.5) and a low BMI (OR per 5 index units more 0.3, 95% CI 0.2–0.7) could be risk factors.

**Fig. 2:**
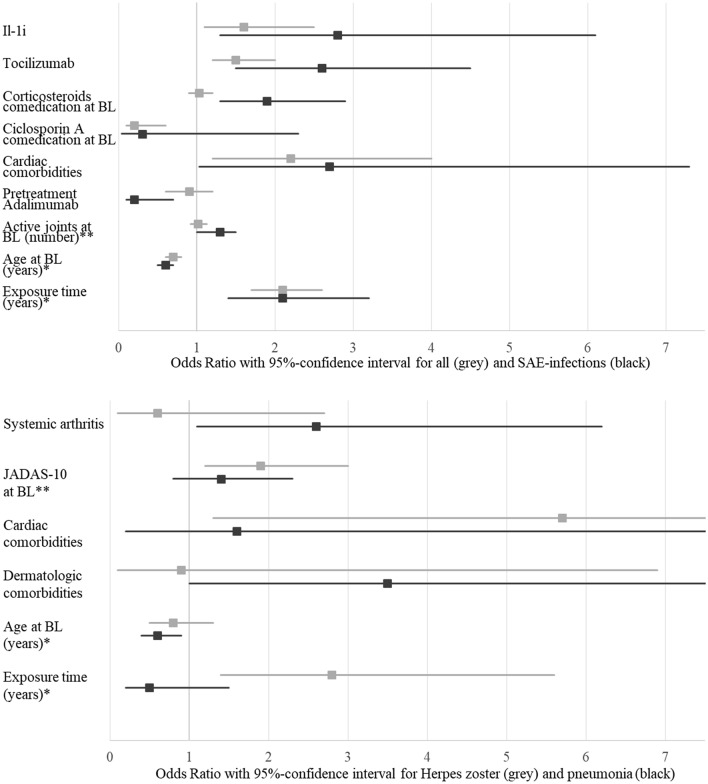
Comparison of covariates regarding all (grey) and infections requiring hospitalization (SAE) (black) in the upper part and Herpes zoster (grey) and pneumonia (black) in the lower part. Only covariates were considered that showed a significant influence on the occurrence of infections in at least one model presented here. *BL* baseline, *IL-1i* Interleukin-1-inhibitors, *JADAS-10* Juvenile Arthritis Disease Activity Score, *5 years per increment of 1, **10 units per increment of 1

A comparative view of the multivariate models for all, SAE and AESI infections is shown in Table 4. The sensitivity and specificity of the predictive models was predominantly above 60%. In addition, we have used Fig. 2 to illustrate how various covariates significantly affect the likeihood of different infections: multivariate analyses also showed an association of treatment with IL-1i or TOC with the occurrence of both common and SAE infections. When considering Herpes zoster, pneumonia and primary varicella, the choice of the biologic is not important in the multivariate models. The presence of cardiac comorbidities is associated with the occurrence of all infections, of SAE infections and the occurrence of Herpes zoster.

**Table 4:** Comparison of multivariate logistic regression for all infections, infections requiring hospitalization (SAE), Herpes zoster, pneumonia and varicella

Covariates	All infections	SAE infections	Herpes zoster	Pneumonia	Varicella
	OR [95% CI]	OR [95% CI]	OR [95% CI]	OR [95% CI]	OR [95% CI]
Interleukin-1-inhibitors	1.6 [1.1/2.5]	2.8 [1.3/6.1]			
Tocilizumab	1.5 [1.2/2]	2.6 [1.5/4.5]			
Systemic arthritis				2.6 [1.1/6.2]	
Corticosteroids comed at BL		1.9 [1.3/2.9]			2.3 [1/5.3]
Ciclosporin A comed at BL	0.2 [0.1/0.6]				
Cardiac comorbidities	2.2 [1.2/4]	2.7 [1/7.3]	5.7 [1.3/25.6]		
Respiratory comorbidities					4.5 [1/20.4]
Dermatologic comorbidities				3.5 [1/11.8]	
Pretreatment Adalimumab		0.2 [0.1/0.7]			
JADAS-10 at BL**			1.9 [1.2/3]		
Number of active joints at BL**		1.3 [1/1.5]			
Age at baseline* [years]	0.62 [0.57/0.69]	0.6 [0.5/0.7]		0.6 [0.4/0.9]	
Exposure time* [years]	2.1 [1.7/2.6]	2.1 [1.4/3.2]	2.8 [1.4/5.6]		2.5 [1.1/5.4]
BMI* [kg/m^2^]					0.4 [0.2/0.8]
Sensitivity of this model	60.3%	58.3%	75%	63.3%	56.5%
Specificity of this model	65.1%	70.3%	68.5%	69.1%	83.3%

*OR* Odds ratio, *CI* confidence interval, *BL* baseline, *IL-1i* Interleukin-1-inhibitors, *comed* comedication, *JADAS* Juvenile arthritis disease activity score, *BMI* Body mass index

*5 years/index units per increment of 1

**10 index units/joints per increment of 1

For all types of infections investigated, the multivariate analyses showed a correlation between younger age at baseline or longer exposure time to the respective biologic and the occurrence of infections.

## Discussion

The German BIKER registry is the largest national registry on the use of biologics in patients with JIA and has accumulated a large quantity of data and observation time. BIKER is covering the whole country with more than 80 participating paediatric rheumatology units and may, therefore, be representative not only for Germany but for a number of comparable countries, while findings could not be extended to other parts of the world. This analysis adds a comparison of the incidence and risk factors of various infectious diseases between different biologics used to treat JIA.

### Common infections

Patients treated with IL-1i were significantly more often affected by infections compared to patients treated with TNFi or ABA. In a randomized multicenter study by Ilowite et al. [20] on patients with pJIA, no significant difference in the incidence of respiratory tract infections was found between patients receiving ANA or placebo. Another placebo-controlled study by Ruperto et al. [21], which focused on the use of CAN in patients with sJIA, showed increased rates of infections in patients receiving CAN. However, when looking at the various studies, it must be taken into account that no comparison with a placebo group was made in the analysis presented here. Rather, the patients treated with IL-1i were compared to patients receiving TNFi. Regarding our analysis, the majority of patients in the IL-1i-cohort had a sJIA, whereas in the TNFi-cohort mainly patients with polyarthritis were found. Interestingly, the significant accumulation of infections in the IL-1i-cohort compared to the TNFi-cohort remains when considering only patients with sJIA. In a 2017 publication on biologics for the therapy of sJIA using data from the BIKER registry, infections were also significantly more frequently observed under therapy with IL-1i than under therapy with ETA [12]. ETA was predominantly used to treat sJIA before 2008 and thereafter was replaced by IL-1i as well as by IL-6i. Patients who received a TNFi to treat sJIA had no lower disease activitiy than patients with sJIA in the IL-1i-cohort. Therefore, it is unlikely that this observation is only existing because of a confounding by different indications.

Patients receiving TOC are also more likely to suffer from an infection. This hypothesis is consistent with two randomized, placebo-controlled studies, one on the safety of TOC in the treatment of sJIA [10] and one in the treatment of pJIA [22]. A Japanese study by Yokota et al. [23] with similar study design as the BIKER registry concludes that 41% of all JIA patients were affected by at least one infection during the first year of treatment with TOC. The proportion of patients with an infection in all patients treated with TOC was 28.2% in our analysis. However, only patients with sJIA were included in the Japanese study. If we also consider only patients with sJIA treated with TOC, the percentage of patients with at least one infection is 38% and thus quite in line with the Japanese study.

### Serious infections

In total, the number of incident serious infections was low with 103 SAE infections (2.8%, IR: 1.2, 95% CI 1/1.5) in 3654 treatment episodes, indicating a surprisingly high safety of biologics considering their immunosuppressive properties. A systematic review regarding 19 trials identified a very similar rate of serious infections in JIA patients. In 810 children treated with biologics, 17 serious infections (2.1%) occurred [24].

Our analysis suggests that the rate of SAE infections under therapy with IL-1i is significantly higher than under therapy with TNFi or ABA. This is also valid when only patients with sJIA are considered. For similar reasons as described above for all infections, this accumulation does not seem to be exclusively due to the high proportion of patients with sJIA in the IL-1i-cohort. In placebo-controlled studies of ANA [20] and CAN [21], only a few infectious SAE were observed without an accumulation among the biologics. Due to the limited comparability with our analysis, the respective results are not necessarily contradictory. The authors of the study on safety of biologics in treatment of sJIA presented above also found an increase in SAE infections under therapy with IL-1i [12].

Our analysis suggests that the use of TOC is associated with a higher rate of SAE infections. Schiff et al. [25] found that the number of infectious SAE seems to correlate with the dose of TOC: the higher the dose, the more both all infections and infections requiring hospitalization. Evidence of such a dose-dependent correlation can also be found in our analysis. A disproportionately high number of SAE infections affect patients with sJIA (Table 2), in whom the dosage interval and thus the monthly dose of TOC is higher than in patients with other JIA categories [10, 22]. However, this analysis cannot be used to determine with certainty whether the described accumulation of infections under high-dose therapy with TOC is due to higher dose or the presence of sJIA. Interestingly, Horneff et al. [26] have observed that compliance of patients with pJIA is highest with TOC compared to ETA and ADA. This argues for a generally good tolerability of TOC, which may be dose-dependent.

### Opportunistic infections

Treatment with IL-1i or TOC does not seem to increase the probability of the occurrence of Herpes zoster and varicella compared to therapy with TNFi or ABA. In this analysis, cases of Herpes zoster occurred exclusively under therapy with TNFi or TOC. This is consistent with the results of two studies, one evaluating the risk of Herpes zoster in patients with JIA [27] and one in adult patients with rheumatoid arthritis [28].

Besides Herpes zoster, two cases of oral candidiasis occurred as opportunistic infections. Active tuberculosis did not occur during our study. According to an analysis on opportunistic infections in patients with JIA using data from the Pharmachild registry, the three most common opportunistic infections are Herpes zoster, tuberculosis and candidiasis. In a total of 8274 patients, 66 cases of Herpes zoster, 10 cases of tuberculosis and 4 cases of oral candidiasis occurred [29]. The rates of Herpes zoster and oral candidiasis are quite in line with the rates we found, while the rate of tuberculosis in the Pharmachild registry seems to be higher. We suggest that this observation is attributable to differences in the general rate of tuberculosis between the countries of Southern and Eastern Europe compared to Germany and other Central European countries. Nevertheless, opportunistic infections in patients with JIA are rare. However, for countries where tuberculosis is endemic, these conclusions cannot be readily transposed. Data from India show that the occurrence of TB in JIA patients treated with biologics is a significant problem there [30].

### Influence of covariates

Looking at the multivariate predictive models, it is noticeable that the diagnosis of sJIA is only represented as a risk factor in one predictive model. The diagnosis of sJIA is conspicuous by its frequency in the univariate comparisons on patients with any infections and on patients with SAE infections. The absence of sJIA as a risk factor in the multivariate models is due to interdependencies of sJIA to treatment with IL-1i and IL-6i. The use of these biologics appears to have greater predictive power regarding the occurrence of infections. Therefore, they were included in the predictive model instead of sJIA.

Cardiac comorbidities are among the factors that stand out when comparing the various multivariate models. They have predictive power for common infections, SAE infections, and Herpes zoster. Various congenital heart diseases can lead to an increase in the frequency of infections [31] as well as in the proportion of severe courses of infection [32]. Our data, therefore, confirm previously published literature on this point.

The influence of systemic corticosteroids on the occurrence of infections is controversially discussed. In adult patients with rheumatoid arthritis, there is evidence that the use of oral corticosteroids is associated with a dose-dependent increasing risk of SAE infections [33]. For children suffering from JIA, Beukelman et al. [34] posit an association between the use of high-dosed systemic corticosteroids and an increased risk of infections leading to hospitalization. Klein et al. [35] found that sJIA patients treated with TOC and systemic corticosteroids had significantly more infections than patients treated with TOC only. Same goes for treatment of pJIA with Adalimumab and systemic corticosteroids compared to treatment with Adalimumab only [36]. Our analysis confirms these results, the use of systemic corticosteroids is shown to be a risk factor for various infections in several of our uni- and multivariate approaches.

A comedication with MTX besides the biologic therapy is often used. According to our analysis, additionally taken MTX does not seem to increase the rate of common or SAE infections. Klein et al. [6] come to similar results when comparing ADA with or without MTX in the therapy of non-sJIA. According to the Dutch JIA registry, switching between biologics is not associated with an increased safety risk [37]. This is in line with our data, a premedication with another biologic agent is not listed as a risk factor for any of the infections investigated here.

According to our analysis, the disease activity of JIA at baseline seems to influence the probability of occurrence of SAE infections and Herpes zoster. In a study from 2016, which also used BIKER data, Becker and Horneff [38] found that increased disease activity of JIA at baseline is an independent risk factor for the occurrence of serious infections. Strangfeld et al. [28] posit an association between higher disease activity of rheumatoid arthritis and the occurrence of Herpes zoster in adults.

All predictive models presented show an association of either younger age at baseline or a higher exposure time to the respective biologic and the occurrence of infections. This is mainly due to methodical reasons, since younger age at baseline comes along with longer observation time in the BIKER registry. However, younger children are generally more susceptible to infectious diseases, as was also found in the STRIVE registry [39].

### Limitations

Our analysis has limitations. ETA has been approved for the treatment of JIA for 19 years, which is considerably longer than other biologics. This is mirrored in an overwhelming proportion of patient numbers and observation years in the TNFi-cohort and must be taken into account when comparing infection rates and OR.

The characteristics of the patients differ in the various cohorts. This means that an increased infection rate cannot be unequivocally attributed to the respective biologic. However, this problem can be partially counteracted by executing multivariate logistic regressions.

Further limitations are the nonrandomized approach arising from a registry setting. Physicians’ decisions may include multiple factors. Especially when considering common infections, the reporting behavior of different physicians may differ. Although the majority of patient files were monitored, under-reporting may occur in some cases.

## Conclusion

In summary, the data confirm observations from controlled trials with biologics in JIA which have been reviewed in 2015 [40]. The safety profiles of actually approved biologics are highly acceptable. However, this analysis shows that both common infections and infections requiring hospitalization are more frequent in JIA patients treated with IL-1i or IL-6i. Further risk factors for the occurrence of both common and serious infections were especially cardiac comorbidities and pre- or concomitant medication with corticosteroids. Patients who exhibit one or more of these characteristics should, therefore, be monitored particularly closely with regard to infections. Thus, the results of this study could help to further improve the safety of JIA therapy with biologics.

## Supplementary Information

Below is the link to the electronic supplementary material.

Supplementary file1 (XLSX 18 KB)

## Data Availability

The datasets generated during and/or analyzed during the current study are available from the corresponding author on reasonable request.
